# 

**Published:** 1881-12-20

**Authors:** 


Entered at the Post-Office at Atlanta as second-class matter.

VOL. XI. DECEMBER 20, 1881. NO. 12.

THE

### SOUTHERN MEDICAL RECORD:

### A MONTHLY JOURNAL OF PRACTICAL MEDICINE.

Terms: $2 00 per Annum, in Advance; 4 Copies $6.00; Single Copies 20 Cents.

*"QUICQUID PRÆCIPIES ESTO BREVIS."*

### WHERE TO BUY.

William R. Warner & Co. .... 1
Celerina .... 2
Petrolina .... 3
J. L. Berg & Co. .... 4-5
Powell's Combined Beef, etc. .... 6
A. A. Mellier .... 7
Sharp & Dohme .... 8
Mellin's Food .... 9
Johnston's Fluid Beef .... 9
Thomas F. Goode .... 10
O. C. St. Clair .... 11
Dundas, Dick & Co. .... 12
Bedford Springs .... 13
Schumann .... 14
N. Y. Pharmacal Association .... 15
Geo. J. Howard & Bro .... 16
Trommer Extract of Malt (ins. front cov.)
Reed & Carnrick .... (inside back cover.)
Parke, Davis & Co. .... (outside back cover.)
McKesson & Robbins .... (colored leaves.)
Parke, Davis & Co .... (colored leaves.)
Scott's Emulsion .... (colored leaves.)
Listerine .... (colored leaves.)
Southern Medical College .... (col. leaves.)
Kidder & Laird .... (colored leaves.)

Address R. C. WORD, M.D., Managing Editor, Atlanta, Ga.

